# The Role of Losartan as a Potential Neuroregenerative Pharmacological Agent after Aneurysmal Subarachnoid Haemorrhage

**DOI:** 10.3390/ijms21186496

**Published:** 2020-09-05

**Authors:** Stefan Wanderer, Lukas Andereggen, Jan Mrosek, Sepide Kashefiolasl, Serge Marbacher, Jürgen Konczalla

**Affiliations:** 1 Department of Neurosurgery, Kantonsspital Aarau, Tellstrasse 25, 5001 Aarau, Switzerland; lukas.andereggen@ksa.ch (L.A.); serge.marbacher@ksa.ch (S.M.); 2 Cerebrovascular Research Group, Department for BioMedical Research, University of Bern, 3008 Bern, Switzerland; 3 Department of Neurosurgery, Goethe-University Hospital, Schleusenweg 2 – 16, 60528 Frankfurt am Main, Germany; jan.mrosek@gmx.de (J.M.); sepide.kashefi@gmx.de (S.K.); j.konczalla@med.uni-frankfurt.de (J.K.)

**Keywords:** aneurysmal subarachnoid haemorrhage, cerebral vasospasm, double-haemorrhage model, cerebrovasculature, endothelin, neuroregeneration, rats

## Abstract

Background: Cerebral vasospasm (CVS) remains a major cause of delayed cerebral ischaemia following aneurysmal subarachnoid haemorrhage (SAH), making it a life-threatening type of stroke with high morbidity and mortality. Endothelin-1 is known as key player mediating a strong vasocontractile effect. Interestingly, losartan restores the impaired vasorelaxative ET(B_1_) receptor function in a non-competitive direct fashion. With this study, we aimed to investigate a potential losartan-dependent vasodilatory effect vice versa by inhibiting NO release through L-NAME, thus pushing forward concepts to alleviate vasospasm and possibly prevent ischaemia and neurodegeneration. Methods: Cerebral vasospasm was induced by the use of an established double-injection rat model. Sprague-Dawley rats were culled on Day 3 after the ictus, and the vasospastic basilar artery was harvested for isometric investigations of the vessel tone. Ring segments were preincubated with and without L-NAME and/or losartan. Results: Preincubation with L-NAME induced dose-dependent vasoconstriction via endothelin-1 in the non-SAH cohort, which was dose-dependently reduced by losartan. After SAH and dose-dependent endothelin-1 administration, maximal contraction was achieved in the control group without losartan. Furthermore, this maximal contraction was significantly decreased in the losartan group and was reversed by L-NAME. Conclusions: After SAH, losartan was shown to positively influence the ET(B_1_) receptor pathway in a non-competitive direct agonistic and indirect fashion. Losartan alleviated the maximum contraction triggered by endothelin-1. This effect was resolved due to NO inhibition by L-NAME. Considering this spasmolytic effect of losartan besides its already well-known effects (attenuating cerebral inflammation, restoring cerebral autoregulation and reducing epileptogenic activity) and alleviating early brain injury, losartan seems to have potential as a promising pharmacological agent after SAH.

## 1. Introduction

Subarachnoid haemorrhage (SAH) due to the rupture of an intracranial aneurysm is a life-threatening type of stroke with high morbidity and mortality that affects, in particular, young working patients [1]. Endothelin-1 is a key player mediating cerebral vasospasm (CVS) [2,3], causing delayed cerebral ischaemia (DCI) [4] and neuronal death. Other factors include cerebral inflammation [5], early brain injury [6], cortical spreading depression [7] and a lack of cerebral autoregulation [8], all impacting the neurological outcome.

The ET(A) receptor, located on smooth muscle cells (SMCs), triggers ET-1 dependent vasoconstriction, whereas the ET(B_1_) receptor, located on endothelial cells (ECs), elicits vasorelaxation [9,10]. ET(B_1_) receptor-mediated vasorelaxation has been shown to be impaired after SAH [11]. After the ictus, the upregulation of 5-hydroxytrypatmine (5-HT)_1B_ receptors as well as the increased expression of angiotensin II type 1 (AT_1_) receptors on the muscular media of the arterial vessels plays an additive role in the development of CVS [12]. Furthermore, the upregulation of AT_1_ receptors in the cerebral arteries plays a leading role in focal cerebral ischaemia, with the blockade of these receptors providing beneficial effects [13]. Like ET-1, angiotensin II (ANG II) acts via two specific receptors, i.e., the AT_1_ and angiotensin II type 2 receptors (AT_2_ receptors). The AT_1_ receptor, located on SMCs, mediates vasoconstriction, whereas the AT_2_ receptor, located on ECs, mediates vasorelaxation [10,14]. Both receptors are activated by ANG II.

Despite intra-arterial spasmolysis in refractory cases [15], the risk of ischaemia and neurodegeneration due to cerebral vasospasm remains high. In this context, losartan (LOS), an AT_1_ receptor antagonist, has shown to possess spasmolytic effects on vessel tone by restoring ET(B_1_) receptor-dependent vasorelaxation after SAH, as well as under physiological conditions [14,16]. This direct agonistic effect of LOS in enhancing vasorelaxation, assessed by addressing all the subparts of the ET(B_1_) receptor pathway—e.g., sarafotoxin S6c (S6c), acetylcholine (ACh), sodium nitroprusside dihydrate (SNP) and cyclic guanosine monophosphate (cGMP)—seemed to be ET(B_1_) receptor-dependent.

The aim of this study was to elucidate the potential LOS-dependent vasodilatory effect on the ET(B_1_) receptor under conditions of NO-release inhibition.

## 2. Results

In total, more than 140 basilar artery ring segments were examined. For the final analysis, 21 sham and 19 subarachnoid haemorrhage segments were included, representing a sample size of six or more segments per subgroup. Neurological deficits were more pronounced after SAH (Table 1). 

The basilar artery diameter was significantly reduced after SAH. Whereas sham-operated vessels had a diameter of 276.13 um ± a standard deviation (SD) of 24.23 um, the basilar artery (BA) segments were significantly reduced after the induction of SAH, at 192.74 ± 20.23 um. More detailed molecular studies were not conducted in this experimental setting, because of a lack of permission in the ethics approval.

### 2.1. Effect of 10^−5^ M L-NAME on ET(A) Receptor-Induced Contraction in Sham Animals Preincubated with (10^−4^ M or 3 × 10^−4^ M) and without LOS

**Figure 1:**
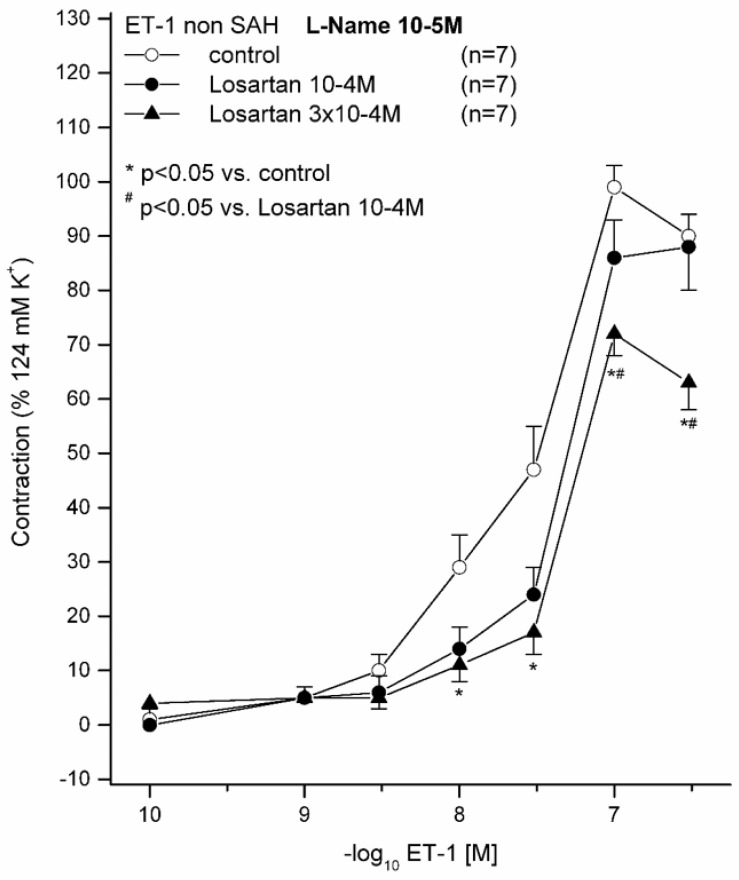
Effect of L-NAME and losartan (LOS) on ET-1-induced contraction in basilar artery ring segments in the sham group. ET-1 induced a dose-dependent contraction. Displayed are the concentration–effect curves (CECs) for each group (with and without LOS). LOS reduced the E_max_ dose-dependently. For the subarachnoid haemorrhage (SAH) with 3 × 10^−4^ M LOS, a statistically significant reduction of E_max_ was calculated. * *p* < 0.05 versus without LOS; # *p* < 0.05 versus 10^−4^ M LOS.

All vessel segments were preincubated with 10^−5^ M L-NAME. ET-1 induced a dose-dependent vasocontraction in all vessel segments by activating the ET(A) receptor, appreciating that the ET(B_1_) receptor-dependent vasorelaxation under physiological conditions is normally masked [3]. For all ET-1 concentrations, contraction was significantly reduced in the 3 × 10^−4^ M LOS group compared to that in the group without LOS (Figure 1). For 3 × 10^−7^ M ET-1, the contraction was also significantly reduced when comparing the 3 × 10^−4^ M LOS with the 10^−4^ M LOS group. Under 3 × 10^−4^ M LOS, the maximum contraction (E_max_) was significantly reduced (73%) compared to that in the group without LOS (99%, *p* = 0.011, pD_2_ (−log_10_EC_50_) = 7.57), whereas the pD_2_ was not significantly altered, indicating that the induced contraction seemed to be affected in a non-competitive way (Figure 1, Table 2). The 10^−4^ M LOS group exhibited an E_max_ of 91%. As the E_max_ was significantly altered in the 3 × 10^−4^ LOS M group, we aimed to test a similar setting under pathophysiological conditions after subarachnoid haemorrhage.

### 2.2. Effects of 10^−5^ M L-NAME with 3 × 10^−4^ M LOS on ET(A) Receptor-Induced Contraction and with (3 × 10^−4^ M) and without LOS on ET(A) Receptor-Induced Contraction in Subarachnoid Haemorrhage

**Figure 2:**
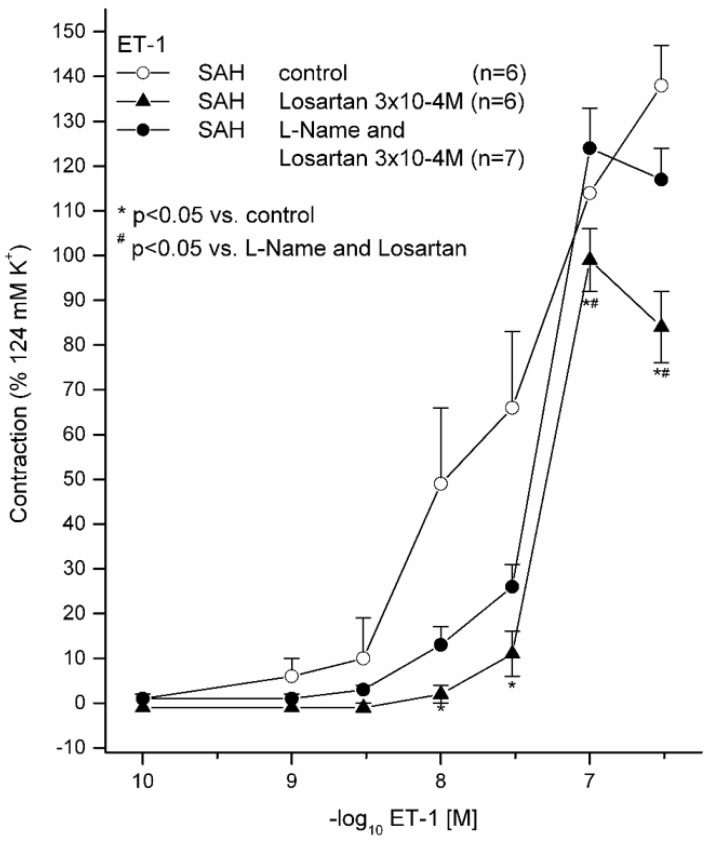
Effect of L-NAME (with and without) and LOS (with and without) on ET-1-induced contraction in basilar artery ring segments in the subarachnoid haemorrhage group. ET-1 induced a dose-dependent vasocontraction. Displayed are the CECs for each group (with and without L-NAME and LOS). LOS without L-NAME reduced the E_max_ dose-dependently. For the SAH with 3 × 10^−4^ M LOS, a statistically significant reduction of E_max_ was calculated. Preincubation with the NO inhibitor L-NAME reduced the vasoreductive effect of LOS. * *p* < 0.05 versus without LOS; # *p* < 0.05 versus L-NAME with LOS.

ET-1 induced, in all the vessel segments, a stronger and higher vasoconstriction, as compared to that in the sham groups (Figure 2, Table 3). For 10^−8^ M ET-1, vasocontraction was significantly reduced in the 3 × 10^−4^ M LOS group compared to that in the 10^−4^ M group; for 3 × 10^−8^ M ET-1, it was significantly reduced in the 3 × 10^−4^ M LOS compared to that in the without-LOS group and in the without-LOS group compared to that in the 10^−5^ M L-NAME with 3 × 10^−4^ M LOS group. With × 10^−7^ M ET-1 3, vasocontraction was significantly reduced in the 3 × 10^−4^ M LOS group compared to that in the group without LOS, and in the 3 × 10^−4^ M LOS group compared to that in the 10^−5^ M L-NAME with 3 × 10^−4^ M LOS group.

**Figure 3:**
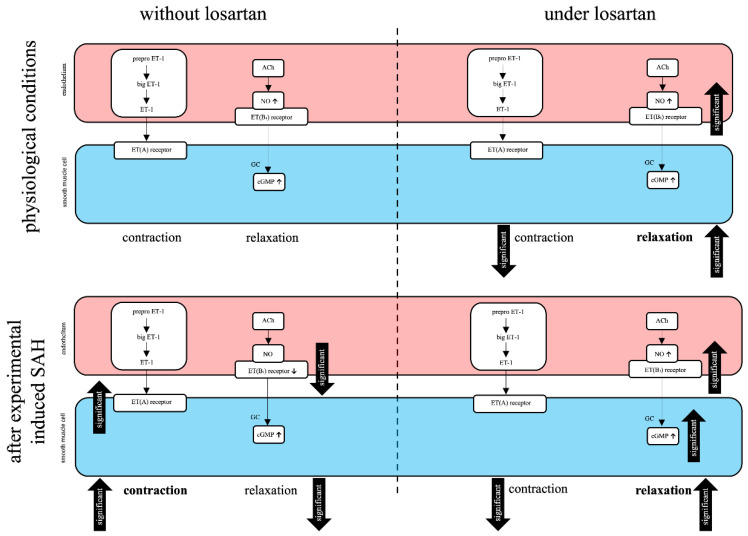
Effects of LOS in the cerebrovasculature under physiological circumstances and after SAH. The upper part of the flow chart describes the physiological circumstances; the lower half, the pathophysiological situation after SAH. The left side describes the physio- and pathophysiological effects in the cerebrovasculature without LOS. An overview of the LOS effects on the cerebrovasculature is displayed on the right side. In each quadrant, the left side describes the contraction, and the right side describes the relaxation pathway. LOS seems to possess a direct effect on the endothelin system, especially on the ET(B_1_) receptor. After SAH, the vasorelaxation mediated by these receptors under physiological conditions is impaired and an increased ET-1 contraction is detected. Under LOS treatment, a significant increase in ET(B_1_) receptor-mediated vasorelaxation was detected, and also, the vasoconstriction mediated by ET-1 was reduced in vessel segments without SAH. After SAH, leading to an increased ET-1 contraction and loss of ET(B_1_) receptor function, under LOS, the ET-1-induced vasoconstriction was reduced and the whole ET(B_1_) receptor-dependent pathway significantly increased.

Segments after SAH without L-NAME and without LOS revealed the highest E_max_ (139%). This vasoconstriction was significantly reduced by 3 × 10^−4^ M LOS preincubation (E_max_, 101%; *p* = 0.016). After preincubation with 10^−5^ M L-NAME, the reduction of E_max_ was abolished (E_max_, 124%; *p* = 0.143; pD_2_ = 7.33). Therefore, NO inhibition by 10^−5^ M L-NAME diminished the vasorelaxative effect of LOS (Figure 3).

## 3. Discussion

Our results show that LOS possesses the ability to restore the vasodilatory ET(B_1_) receptor function after SAH, even under the concomitant inhibition of NO release by L-NAME.

It is commonly known that SAH due to aneurysm rupture causes the functional inactivation of the ET(B_1_) receptors [11]. Under physiological conditions, LOS revealed a dose-dependent antagonistic effect on ET-1-mediated vasoconstriction. This effect was abolished after preincubation with a selective ET(B_1_) receptor antagonist. Moreover, with LOS, increased ET(B_1_) receptor-dependent vasorelaxation than that with S6c, a direct ET(B_1_) receptor agonist, was detected [14]. After subarachnoid haemorrhage, the ET-1-induced vasoconstriction was decreased in parallel by preincubation with LOS and abolished after preincubation with a selective ET(B_1_) receptor antagonist. In precontracted vessels, in the presence of LOS paired with an ET(A) receptor antagonist, ET-1 induced greater vasorelaxation [16]. Further functional investigations confirmed this fact by the significantly enhanced vasodilatation of the whole ET(B_1_) receptor pathway (S6c, ACh, SNP and cGMP). For all parts of the receptor pathway, a clear and consequently significant improvement in vasorelaxation was observed. In this context, data supporting the vasorelaxant effect of LOS under physiological circumstances have already been published by our group: under physiological conditions, S6c already induced a dose-dependent vasorelaxation of precontracted vessel segments. For 3 × 10^−4^ M LOS, the concentration–effect curve (CEC) was significantly shifted to the left, and the E_max_ became significantly higher. ACh, as well, triggered a dose-dependent vasorelaxation. Between 10^−5^ M, 10^−4^ M and 3 × 10^−4^ M LOS, no significant differences in vasorelaxation were calculated. SNP also induced a dose-dependent relaxation after prior precontraction of the BA segments. Here, for 3 × 10^−4^ M LOS, at all concentrations of SNP except 10^−3^ M, statistically significantly higher vasorelaxations were calculated. Furthermore, the pD_2_ was significantly shifted to the left, implementing an earlier vasorelaxation to LOS. cGMP also induced a dose-dependent vasorelaxation in precontracted vessels. For 3 × 10^−4^ M LOS, a trend to higher relaxation was calculated but neither E_max_ nor pD_2_ were significantly changed [14]. Similar results were obtained under pathophysiological conditions after SAH; S6c induced a dose-dependent vasorelaxation. For 3 × 10^−4^ M LOS, the CEC was significantly shifted to the left, with a higher E_max_. ACH induced a dose-dependent vasorelaxation, as well. For 3 × 10^−4^ M LOS, the E_max_ was significantly higher. SNP also induced a dose-dependent relaxation. For 3 × 10^−4^ M LOS, almost all concentrations of SNP induced a statistically higher vasorelaxation. An earlier vasorelaxation was observed, as already confirmed in the sham group. cGMP induced a dose-dependent relaxation, too. For 3 × 10^−4^ M LOS, a significant higher and earlier relaxation was calculated [16]. All these investigations, including the acquisition of the presented data, were performed without ANG II in the organ bath, suggesting the non-competitive ET(B_1_) receptor antagonism of LOS.

There is a direct agonistic enhancing ET(B_1_) receptor-dependent pathway of LOS, so we wanted to elucidate whether this effect could be confirmed vice versa by NO inhibition through L-NAME. Under physiological conditions, these findings were clearly confirmed, as L-NAME inhibited NO release, with an E_max_ of 99% in the without-LOS group. This E_max_ was found to be clearly higher than that with L-NAME inhibition in the 10^−4^ M (91%) and 3 × 10^−4^ M (73%) LOS groups. Thus, the dose-dependent impairment of the ET(B_1_) receptor pathway under LOS combined with L-NAME is obvious. Since the experiments in the sham group were not performed without LOS and with L-NAME, a literature research revealed fairly similar contractile responses in mesenterial arteries without LOS and L-NAME as compared to our results [17].

Compared to in the sham group, in the subarachnoid haemorrhage group, a higher and expected increased sensitivity to ET-1-mediated contraction was detected. A similar effect was also identified in the same experimental setting under LOS [14,16]. Under preincubation with 10^−5^ M L-NAME and with 3 × 10^−4^ M LOS in subarachnoid haemorrhage vessels (E_max_ 124%), the diminished vasoconstriction (E_max_ 101%) with 3 × 10^−4^ M LOS compared to that in the control group without (139%) was abolished, indirectly confirming an ET(B_1_) receptor-dependent effect of LOS as well.

### 3.1. The Role of LOS in a Translational Context

LOS is already well known to possess multiple protective effects in stroke and after subarachnoid haemorrhage. While reactivating the functional impaired ET(B_1_) receptor and thus enhancing vasorelaxation [14,16], it diminishes cerebral inflammation in a physiological and pathophysiological manner after subarachnoid haemorrhage [18]. It attenuates epileptogenic activities [19] and therefore could also be applied in preventing spreading depressions after SAH. LOS is further able to restore cerebral blood flow (CBF) and pressure-dependent vasoconstriction [20]. Moreover, a recent systematic review revealed the beneficial effects of sartans, also considering LOS, on ischemic stroke in both preclinical and clinical settings by attenuating ischemic brain damage, reducing cerebral inflammation and infarct size and increasing CBF [21]. Telmisartan, for example, robustly supported neuroregeneration in a middle cerebral artery occlusion model in rats by downregulating caspase activation [22]. LOS administration in a rat model was also shown to attenuate next-seizure activity and neuronal damage without affecting behavioural changes in a model of hypertension and epilepsy. LOS attenuated epileptogenesis and exerted neuroprotection in the hippocampal CA1 region. This effect could be assessed in long-term experiments after kainite-induced status epilepticus. Likewise, this could be tested regarding behavioural as well as biochemical changes and neuronal damage in a model of hypertension and epilepsy. In that study, LOS induced neuroprotection after 16 weeks, mostly in the hippocampal CA3 region and in certain parts of the dentate gyrus in spontaneously hypertensive rats [23].

SAH following aneurysm rupture, considering strokes in general, accounts for only 5% but can occur at a fairly young age in working people [1,24]. Thus, with LOS administration in these patients, CVS with DCI and DIND could be prevented. At the very least, the extent of DCI may be reduced by LOS [25]. In other words, a LOS-based medical approach after SAH could prevent neurodegeneration by antagonising CVS at an early stage. The current literature shows promising results concerning this issue [21].

### 3.2. Further Perspectives

Considering all these positive effects of sartans [21], especially of LOS by reducing epileptogenicity [19,23,26], cerebral inflammation [18] and infarct size [27] and promoting neuroregeneration [23], this drug might be a valuable option as a neuroprotectant when administered systemically in patients with acute-onset SAH over and above the phase of CVS. Furthermore, it restores the impaired ET(B_1_) receptor function [16] and antagonises vasoconstrictive AT_1_ receptors, which are highly expressed after SAH [12]. Particularly, LOS seems not to influence the global CBF in essential-hypertonic patients. This circumstance could be set equivalent to a needed hypertonia in patients after aneurysm rupture [28]. CVS, cerebral inflammation, the area of the infarct and cortical spreading depression might be reduced.

## 4. Material and Methods

### 4.1. Animals

Male Sprague-Dawley rats, each 310–420 g in body weight (mean, 362.16 g ± 13.12), were included in this study after completing the required acclimatisation period. The ARRIVE guidelines were strictly followed [29]. Preoperatively, a maximum of 5 rats were kept in a cage with a 12 h dark–light cycle and food and water ad libitum. Rats were randomised to either the sham or bleeding group; blinding was not possible due to the postoperative neurological deficits in the SAH group. All the operative procedures were performed with the permission of the local ethics committee (Gen. Nr. F 138/12, 28 December 2009, Regierungspräsidium Darmstadt, Germany) and in compliance with the German Federal Guidelines for animal experiments.

### 4.2. Anaesthesia and Surgical Protocol

Anaesthesia was performed with midazolam (1 mg/kg of body weight, intraperitoneal) and ketamine (100 mg/kg of body weight, intraperitoneal). Afterwards, an inguinal incision was performed with dissection of the femoral artery, vein and nerve. Afterwards, spontaneously breathing rats were fixed in a stereotactic frame. A suboccipital incision was performed, and the atlantoocipital membrane was opened up. With a small syrinx and silicone tube fixed, 0.1 mL of cerebrospinal fluid was withdrawn, and then afterwards, 0.2 mL of autologous blood was injected into the cisterna magna to mimic bleeding. Wounds were closed in a standardised fashion, and the same surgical procedure was repeated 24 h later. The sham group received an injection of a 0.9% isotonic sodium chloride solution, twice. During the postoperative phase, all the animals received 5 mL of crystalloid solution with 0.0125 mg of fentanyl (Janssen-Cilag Pharma GmbH, Neuss, Germany) intraperitoneally at least twice a day as pain medication. The severity of bleeding induction, including neurological deterioration, angiographic vasospasm and MR-perfusion deficits, was already published [30]. However, due to reduction of pain, angiography and MR perfusion were not performed in these settings, but long-lasting neurological deterioration was determined in every SAH-induced rat.

### 4.3. Further Processing of Brain Vessels

**Figure 4:**
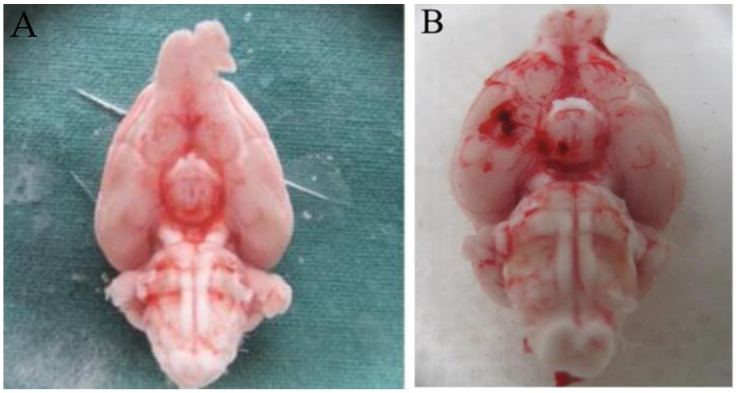
Macroscopic assessment of the brain in the sham and SAH groups. (**A**) shows the sham brain; no signs of subarachnoid bleeding patterns were detected. (**B**) shows the SAH brain with clear blood clots in the basal cisterns.

On Day 3 postoperatively, all the animals were deeply anesthetised by using CO_2_ and afterwards sacrificed by exsanguination after cutting the external and internal carotid arteries. The brain, along with the cerebral vessel complex, was carefully excised and immediately immersed in cold modified Krebs–Högestätt solution, freshly prepared on the day of the experiment. Prior to euthanasia, SAH was confirmed by manifest neurological deficits, assessed on a neurological grading scale [31]. SAH was confirmed by macroscopic inspection of the brain (Figure 4), with the confirmation of blood clots in the basal cisterns. The basilar artery (BA) together with a small subarachnoid plane was carefully dissected from the brainstem by using a binocular microscope (Carl Zeiss, Oberkochen, Germany). Each BA was cut into four equal parts, measuring 2 millimetres (mm) in length. All the ring segments were carefully mounted on L-shaped stainless steel rods and installed in an organ bath (IOA-5301; FMI GmbH, Seeheim-Ober Beerbach, Germany). Isometric force changes were measured in millinewtons (mN) and recorded by using a transducer (GM Scaime, Annemasse Cedex, France). All the rats and BA segments were randomly assigned to different experimental groups, allowing for the use of vessel segments from different rats in the final analysis of each group.

### 4.4. Experimental Setting

Organ baths were filled with a modified Krebs–Högestätt solution and continuously purged with a gas mixture (95% O_2_, 5% CO_2_), resulting in a humidified pH of approximately 7.35. For the investigations, 21 sham and 19 subarachnoid haemorrhage segments were analysed. Vessel segments without LOS were defined as the control group. A reference contraction was induced by 124 mM potassium+ Krebs solution (124 mM KCl) (Krebs–Högestätt solution with an equimolar exchange of sodium chloride (NaCl) with KCl), which was repeated at the end of the experiment. Vessel segments reaching less than 2 mN contraction were excluded before starting the experimental protocol, and vessel segments developing less than 75% of the initial reference contraction at the end of the experiment were also excluded. The functional integrity of the endothelium was tested by the administration of ACh (10^−4^ M) after precontraction with 5-HT (10^−5^ M). A vasorelaxation of more than 30% of the precontraction indicated a functionally intact endothelium. Segments not reaching this percentage were excluded from further assessment.

After one hour, L-NAME was added in the organ bath for all sham segments at 10^−5^ M, and in the subarachnoid haemorrhage segments, it was added only for the 3 × 10^−4^ M LOS group, followed by an incubation period of 15 min. LOS was added in the organ bath for the sham group at 10^−4^ M and 3 × 10^−4^ M and in the subarachnoid haemorrhage group at 3 × 10^−4^ M, and they were compared to the corresponding solvent-control groups. In each case, only for one LOS concentration a CEC was performed by cumulative application to avoid tachyphylaxis. After a 30 min incubation period with or without LOS, concentration–effect curves (CECs) for ET-1 (10^−10^ to 3 × 10^−7^ M) were generated by cumulative application. A more detailed protocol has been published in a previous publication [32]. The LOS-concentration range was determined after preliminary studies, as described by analysing the angiotensin- and endothelin-crosstalk in prior publications [14]. Based on the data from Gurzu et al. and pre-tests for prior publications, this concentration range was also used in this setting [14,33].

### 4.5. Compounds and Solvents

Krebs–Högestätt solution was composed of the following components: NaCl (Sigma Aldrich, Schnelldorf, Germany), 119 mM; KCl (Sigma Aldrich, Germany), 3.0 mM; sodium dihydrogen phosphate, 1.2 mM (AppliChem, Darmstadt, Germany); calcium chloride, 1.5 mM (AppliChem, Germany); magnesium chloride (Merck, Darmstadt, Germany), 1.2 mM; sodium hydrogen phosphate (VWR International BVBA, Oud-Heverlee, Belgium), 15 mM; and glucose (Sigma Aldrich, Germany), 10 mM.

5-HT, ACH, ET-1, L-NAME and LOS were purchased from Sigma-Aldrich (Schnelldorf, Germany). All compounds were freshly dissolved in distilled water on the day of the experiment. The choice of these compounds was explained previously in detail [34].

### 4.6. Analysis of Results and Statistics

Contraction was measured in mN and was given as a percentage of the reference contraction. All the values in the text and figures are given as mean ± SD. For each completed CEC, the E_max_ and pD_2_ or EC_50_ (i.e., the concentration at which half of the maximal effect occurs) were calculated.

All statistical analyses were performed using one-way analysis of variance followed by the Scheffe test for the post hoc comparison of mean values. A probability value (*p*) less than 0.05 was considered significant. Data were analysed using IBM SPPS^®^ (version 22, USA). The sample size per group was determined using an a priori sample size calculation (BiAS.for.Windows™^®^ Version 11, EPSiLON Verlag, Germany). To achieve *α*  =  0.05 at *β*  =  0.2 with a sigma of 0.2, the sample size calculation showed that *n*  =  4–8 segments per group was appropriate to have a delta between 0.3 and 0.5. The figures were visualised using Microcal Origin 7.0 (OriginLab, Northampton, MA, USA).

## 5. Conclusions

In summary, the application of LOS was furthermore confirmed to be associated with an indirect restorative effect on the functionally impaired ET(B_1_) receptors. Considering all the positive aspects of this already clinically well-established pharmacological agent, it might have a role as an underrated neuroprotective drug in the further treatment of SAH patients. In order to apply it in clinics, the efficacy of LOS should be assessed in in vivo SAH models as well as in further ex vivo systems. To determine its potential value after SAH, further animal studies with more detailed molecular investigations as well as multicentric prospective clinical trials are warranted.

## Figures and Tables

**Table 1:** Neurological deficits were evaluated using the Bederson Scale. Normal was judged as both forepaws reaching out (0); mild, as flexion of the contralateral limb (1); moderate, as decreased resistance to lateral push without circling (2); and severe, as decreased resistance to lateral push with circling (3).

	Sham (*n =* 21)	SAH (*n =* 19)
**Normal**	20	0
**Mild**	1	4
**Moderate**	0	8
**Severe**	0	7

**Table 2:** Contractile assessment (ET-1) after incubation with L-NAME, a NO-inhibitor, without and with LOS, an AT_1_ receptor antagonist, under physiological conditions. ET-1 induced a dose-dependent contraction, which was significantly reduced by LOS. This LOS-dependent effect was dose-dependent as well. Values are expressed as mean ± SD. * *p* < 0.05 versus without LOS.

	ET-1	E(max) Contraction	pD_2_	*n*
**Sham with 10^−5^ M L-NAME without LOS**		99 ± 11%	7.57 ± 0.30	7
**Sham with 10^−5^ M L-NAME and 10^−4^ M LOS**		91 ± 19%	7.35 ± 0.08	7
**Sham with 10^−5^ M L-NAME and 3 × 10^−4^ M LOS**		73 ± 12% *	7.35 ± 0.08	7

**Table 3:** Contractile assessment (ET-1) after preincubation with L-NAME and LOS, as well as after preincubation with and without LOS only under pathophysiological conditions after subarachnoid haemorrhage. ET-1 induced a dose-dependent contraction, which was significantly reduced under LOS. This LOS-dependent effect was less pronounced after prior NO inhibition through L-NAME. Values are expressed as mean ± SD. * *p* < 0.05 versus without LOS.

	ET-1	E(max) Relaxation	pD_2_	*n*
**SAH without LOS**		139 ± 22%	7.64 ± 0.52	6
**SAH with LOS 3 × 10^−4^ M**		101 ± 12% *	7.28 ± 0.06	6
**SAH with L-NAME 10^−5^ M and LOS 3 × 10^−4^ M**		124 ± 24%	7.33 ± 0.04	7
