# Correction: Wanderer et al. The Role of Losartan as a Potential Neuroregenerative Pharmacological Agent after Aneurysmal Subarachnoid Haemorrhage. *Int. J. Mol. Sci.* 2020, *21*, 6496

**DOI:** 10.3390/ijms26052312

**Published:** 2025-03-05

**Authors:** Stefan Wanderer, Lukas Andereggen, Jan Mrosek, Sepide Kashefiolasl, Serge Marbacher, Jürgen Konczalla

**Affiliations:** 1 Department of Neurosurgery, Kantonsspital Aarau, Tellstrasse 25, 5001 Aarau, Switzerland; lukas.andereggen@ksa.ch (L.A.); serge.marbacher@ksa.ch (S.M.); 2 Cerebrovascular Research Group, Department for BioMedical Research, University of Bern, 3008 Bern, Switzerland; 3 Department of Neurosurgery, Goethe-University Hospital, Schleusenweg 2 – 16, 60528 Frankfurt am Main, Germany; jan.mrosek@gmx.de (J.M.); sepide.kashefi@gmx.de (S.K.); j.konczalla@med.uni-frankfurt.de (J.K.)

## Error in Figure

In the original publication [1], there was a mistake in Figure 4A as published. Unintentionally, the authors chose the same image as that published in Acta Neurochir (Wien). November 2016;158(11):2075–2083. Doi:10.1007/s00701-016-2939-5. Epub 10 September 2016. The corrected Figure 4A appears below. The authors state that the scientific conclusions are unaffected. This correction was approved by the Academic Editor.

The authors apologize for the mistake and declare again that the conclusion is accurate.

**Figure 4:**
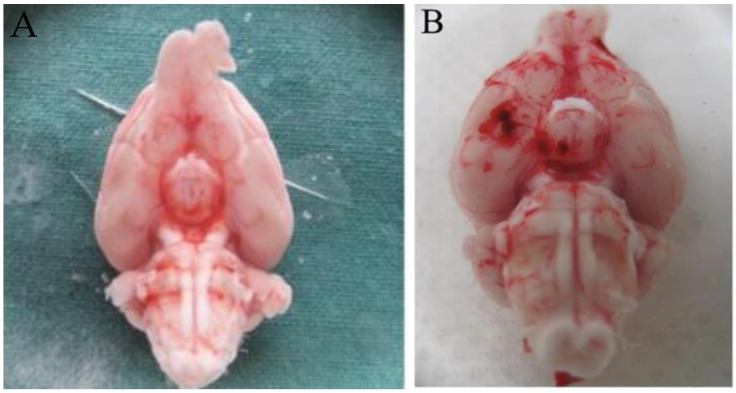
Macroscopic assessment of the brain in the sham and SAH groups. (**A**) shows the sham brain; no signs of subarachnoid bleeding patterns were detected. (**B**) shows the SAH brain with clear blood clots in the basal cisterns.
